# Assessing public health service capability of primary healthcare personnel: a large-scale survey in Henan Province, China

**DOI:** 10.1186/s12913-024-11070-4

**Published:** 2024-05-14

**Authors:** Rongmei Liu, Qiuping Zhao, Wenyong Dong, Dan Guo, Zhanlei Shen, Yi Li, Wanliang Zhang, Dongfang Zhu, Jingbao Zhang, Junwen Bai, Ruizhe Ren, Mingyue Zhen, Jiajia Zhang, Jinxin Cui, Xinran Li, Yudong Miao

**Affiliations:** 1 Henan Key Laboratory for Health Management of Chronic Diseases, Central China Fuwai Hospital, Central China Fuwai Hospital of Zhengzhou University, Zhengzhou, Henan China; 2 Department of Hypertension, Henan Provincial People’s Hospital, People’s Hospital of Zhengzhou University, Henan, China; 3 Department of Health Management, College of Public Health, Zhengzhou University, No.100 Kexue Road, Zhongyuan District, Zhengzhou, Henan 450001 China; 4 Department of Neurology, Henan Provincial People’s Hospital, People’s Hospital of Zhengzhou University, Henan, China

**Keywords:** Primary healthcare personnel, Public health service capability, Urban and rural areas

## Abstract

**Background:**

The public health service capability of primary healthcare personnel directly affects the utilization and delivery of health services, and is influenced by various factors. This study aimed to examine the status, factors, and urban-rural differences of public health service capability among primary healthcare personnel, and provided suggestions for improvement.

**Methods:**

We used cluster sampling to survey 11,925 primary healthcare personnel in 18 regions of Henan Province from 20th to March 31, 2023. Data encompassing demographics and public health service capabilities, including health lifestyle guidance, chronic disease management, health management of special populations, and vaccination services. Multivariable regression analysis was employed to investigate influencing factors. Propensity Score Matching (PSM) quantified urban-rural differences.

**Results:**

The total score of public health service capability was 80.17 points. Chronic disease management capability scored the lowest, only 19.60. Gender, education level, average monthly salary, professional title, health status, employment form, work unit type, category of practicing (assistant) physician significantly influenced the public health service capability (all *P* < 0.05). PSM analysis revealed rural primary healthcare personnel had higher public health service capability scores than urban ones.

**Conclusions:**

The public health service capability of primary healthcare personnel in Henan Province was relatively high, but chronic disease management required improvement. Additionally, implementing effective training methods for different subgroups, and improving the service capability of primary medical and health institutions were positive measures.

**Supplementary Information:**

The online version contains supplementary material available at 10.1186/s12913-024-11070-4.

## Background

Public health services play a pivotal role in safeguarding people’s health and shaping their future. In this domain, primary healthcare personnel are the direct providers of essential public health services and the backbone of China’s primary health care [1]. They deliver various public health services, such as health lifestyle guidance, chronic disease management, health management of special populations, and vaccination services. Studies have shown that the implementation of the national basic public health service project [2, 3], which is one of the important components of China’s basic medical and health service system [4], has successfully achieved its intended outcome [5]. This initiative not only effectively manages the risk factors affecting the population, leading to a decrease in the occurrence of common and chronic diseases, but also improves the overall health quality of the population [6, 7].

However, the public health service capability of primary healthcare personnel may be difficult to cope with the growing demands and challenges [8, 9] of public health service projects. As public health service projects gradually advance, China’s primary healthcare personnel face more and more requirements [10]. Hence, there is a pressing need to evaluate their current public health service capability [11]. However, the current research on primary healthcare personnel mainly focuses on personnel training itself [12], while there are few studies based on the capability of basic public health service projects. In addition, in the existing studies, the public health service capability is embodied in the medical and health institutions [13], and there is a lack of comprehensive evaluation of the service capability of primary healthcare personnel. This assessment is critical for accelerating the progressive equalization of basic public health service projects, strengthening the development of a more comprehensive public health service system [14], and ultimately elevating the overall health status of the population.

The public health service capability of primary healthcare personnel may be influenced by various factors, such as gender, age, profession, region, etc [15]. . . Studies have reported that primary health institutions face many problems [16] such as excessive work pressure [17, 18], uneven distribution of medical and health resources [19], and service quality [20] among the primary healthcare personnel. Furthermore, some studies have suggested that primary healthcare personnel possess limited theoretical knowledge and practical skills [21] in public health service capability. These limitations hinder the effective implementation and widespread adoption of basic public health services [22]. Moreover, primary healthcare personnel may experience different levels of job burnout [23, 24] due to heavy workloads and excessive temporary assignment by their work. There may also be regional differences in the delivery of public health services between urban and rural areas [25, 26], which may affect the public health service capability of primary healthcare personnel.

Henan Province, an important province in the Central Plains of China with a large primary population and great medical needs, is a pioneer of primary public health service reform. Therefore, this study chose Henan Province as the research site, which helped to reflect the current status, influencing factors, and formation mechanism of the public health service capability of primary healthcare personnel in China. It also analyzed the differences in basic public health services between urban and rural areas, explored ways to improve the public health service capability of primary healthcare personnel, and provides empirical support for the sustainable development of basic public health services.

## Methods

### Participants and study procedure

**Fig. 1:**
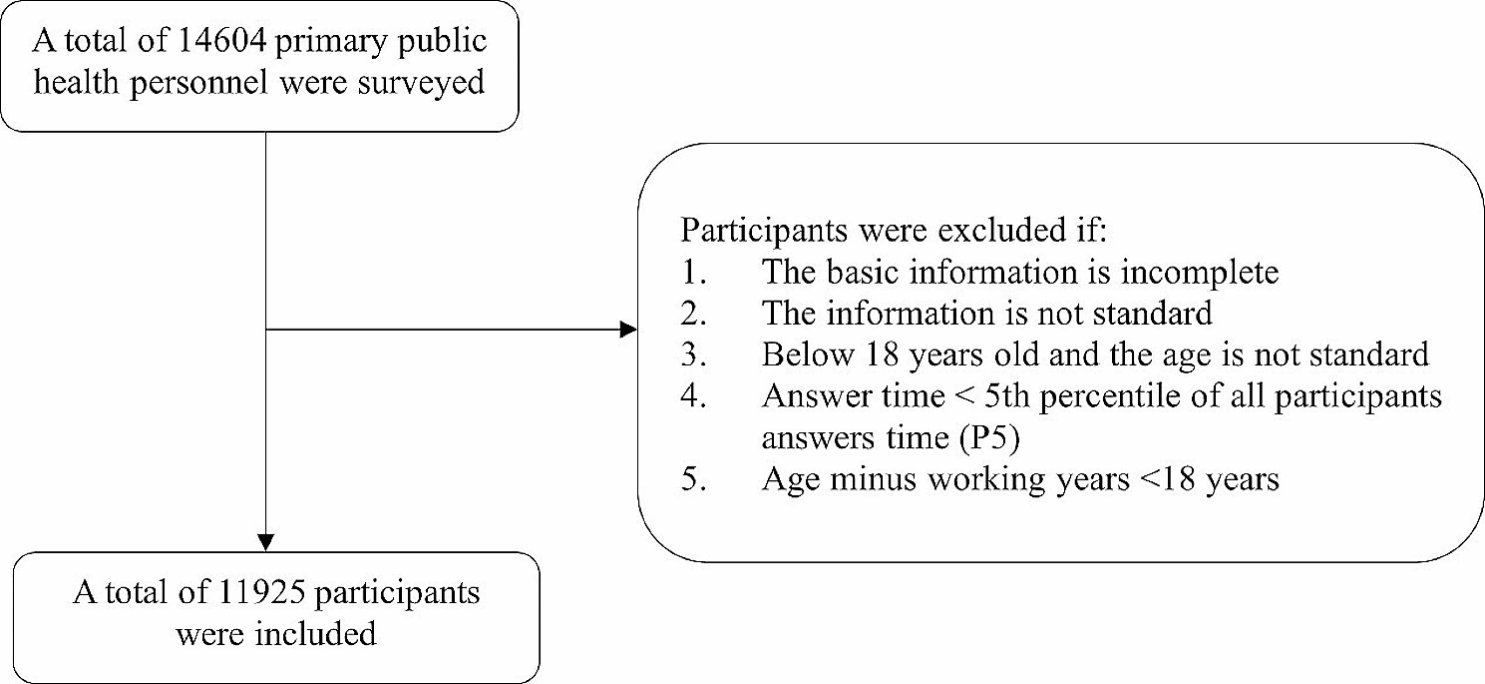
Inclusion and exclusion of participants

We conducted a cross-sectional survey of primary healthcare personnel in 18 cities of Henan Province, China, one of the most populous and important provinces in central China. The survey period was from March 20, 2023 to March 31, 2023. A self-designed questionnaire was used to collect data, mainly through the municipal disease prevention and control, community health service centers, and township health centers in Henan Province. And a total of 14,604 questionnaires were collected, excluding those with incomplete basic information and irregular information filling, those under 18 years old, irregular age filling, short response time and those with age minus working experience less than 18 years. After screening, there were 11,925 valid questionnaires, with an effective recovery rate of 81.66%. The inclusion and exclusion process were shown in Fig. 1.

### Assessment

Based on the World Organization of Family Doctors (WONCA) tree model [25] and the European Quality and Cost of Primary Care (QUALICOPC), we designed a questionnaire for primary healthcare personnel [26], which is widely recognized internationally and has high scientific and reliability. At the same time, according to the structure of national basic public health service project [27, 28], the regional characteristics, population structure and medical and health service needs of Henan Province, some indicators in the questionnaire were added, deleted and adjusted to design the questionnaire (Supplementary file 1). After expert consultation and review, a preliminary survey was conducted in two communities and two townships within Zhengzhou to assess the reliability and validity of the questionnaire. The scale consists of two parts: basic information of primary healthcare personnel and public health service capability rating scale. The Cronbach’s α coefficient of the scale was 0.980, and the KMO test value was 0.973, indicating good reliability and validity of the scale.

#### Basic information

The first part of the questionnaire collected basic information of the participants, such as gender, age, marital status, education level, health status, working area, working years, forms of employment, type of work unit, category of practicing (assistant) physicians, professional title, average monthly salary.

#### Dependent variable

The second part of the questionnaire measured the public health service capability of the participants. It had four dimensions: (1) healthy lifestyle guidance capability, including guidance capability of diet, exercise, decompression, weight, smoking cessation, alcohol limit; (2) chronic disease management capability, including chronic disease screening, risk prediction, integrated chronic disease management, management effect evaluation; (3) health management capability of special populations (It refers to children aged 0–6 years, pregnant women, the elderly, patients with hypertension, or type 2 diabetes, etc.), including professional and technical training, information mastery, health education, health records capability; and (4) vaccination service capability, including receive vaccination training, understand immunization programmer, vaccination procedures, vaccine alternatives, safety precaution, etc.

Using a five-point likert scale, the options for each item were “strongly disagree”, “disagree”, “neutral”, “agree”, and “strongly agree”, scored from 1 to 5 respectively. The score of each dimension ranged from 5 to 25, and the overall score ranged from 20 to 100. A higher score indicated a higher level of public health service capability.

### Statistical analysis

The means with standard deviation was used to describe the continuous quantitative variables and percentages was used to present the categorical variables. Analysis of variance (ANOVA) and student’s t- test were used to test the difference in mean scores among the groups. Collinearity testing was employed to examine the relationships between independent variables. The results revealed no significant collinearity among the independent variables, as indicated by variance inflation factors (VIF) below 5. Multivariate linear regression was used to analyze the influencing factors of primary public health service capability. The Propensity Score Matching (PSM) was used to analyze the differences in the public health service capability of primary healthcare personnel between urban and rural areas.

## Results

### Baseline characteristics of primary healthcare personnel

The study included 11,925 primary healthcare personnel. Of these, 296 (2.48%) did not meet the required criteria, while 4,175 individuals (35.02%) demonstrated excellent public health service capacities. The table shows the score of public health service capability of primary healthcare personnel in Henan Province as 80.17 (Table 1). Male public health service capability score was 81.21 points, 1.8 points higher than female. The primary healthcare personnel aged 51–60 had a mean score of 80.71, while those with 21 years of work experience had a mean score of 80.83 points. The public health service score of high school education and below was 80.42 points. Public health services with the average monthly salary of 3001–4500 yuan scored 80.80. The score of intermediate professional title was 80.80, and the score of healthy people was 80.55. The score of those who were regular employee was 80.65, and the village clinic was 81.19. Within the category of practicing or assistant physicians, public health and clinical category scored higher (Table 1).

**Table 1:** Characteristics of the study participants in association to the public health service capability scores

Variables	Number of Participants (%)	Public health service capability score (M ± SD)	*P*-value
Population	11,925	80.17 ± 14.12	
Gender			
Female	6889 (57.77)	79.41 ± 14.00	< 0.001†
Male	5036 (42.23)	81.21 ± 14.21	
Age			
18–30	1210 (10.15)	79.07 ± 15.75	0.001‡
31–40	2941 (24.66)	79.81 ± 14.36	
41–50	4073 (34.16)	80.17 ± 13.87	
51–60	3022 (25.34)	80.71 ± 13.43	
≥ 61	679 (5.69)	81.17 ± 14.11	
Marital status			
Married	10,984 (92.11)	80.27 ± 14.00	0.026†
Others	941 (7.89)	79.11 ± 15.42	
Education level			
High school and below	6184 (51.86)	80.42 ± 13.88	0.066‡
Junior college	3770 (31.61)	79.75 ± 14.29	
Bachelor degree and above	1971 (16.53)	80.21 ± 14.50	
Working years			
0–5	2207 (18.51)	79.25 ± 14.92	< 0.001‡
6–10	1863 (15.62)	79.55 ± 14.47	
11–15	1268 (10.63)	80.15 ± 14.19	
16–20	1343 (11.26)	80.04 ± 13.88	
≥ 21	5244 (43.97)	80.83 ± 13.65	
Average monthly salary			
3000 and below	8546 (71.66)	79.88 ± 14.14	0.001‡
3001–4500	2915 (24.44)	80.80 ± 14.18	
4501 and above	464 (3.89)	81.59 ± 12.98	
Professional title			
No title	3734 (31.31)	79.72 ± 14.06	0.031‡
Primary title	5556 (46.59)	80.19 ± 14.20	
Intermediate title	2208 (18.52)	80.80 ± 13.83	
Senior title	427 (3.58)	80.75 ± 14.94	
Health status			
Health	10,275 (86.16)	80.55 ± 14.14	< 0.001‡
Sub-health	1197 (10.04)	77.03 ± 13.84	
Disease	453 (3.8)	79.93 ± 13.30	
Forms of employment			
Others	3160 (26.5)	80.18 ± 13.62	0.002‡
Contracted employee	4402 (36.91)	79.50 ± 14.41	
Regular employee	4363(36.59)	80.65 ± 14.37	
Type of work unit			
Village clinic	5608 (47.03)	81.19 ± 13.33	< 0.001‡
Community health service centers (stations)	1825 (15.3)	79.50 ± 14.07	
Township health center	4492 (37.67)	79.18 ± 14.98	
Category of practicing (assistant) physician			
Public health category	6255 (52.45)	80.19 ± 14.26	0.049‡
Clinical category	4026 (33.76)	80.29 ± 13.85	
Traditional Chinese medicine category	1499 (12.57)	80.11 ± 14.08	
Oral category	145 (1.22)	76.95 ± 15.38	

P† value from t-test; P‡ value from analysis of variance

In addition, there were statistical differences in the public health service capability scores of primary healthcare personnel among different groups, such as gender, age, marital status, working years, average monthly salary, professional title, health status, form of employment, type of work unit, category of practicing (assistant) physicians (All *P* < 0.05).

**Fig. 2:**
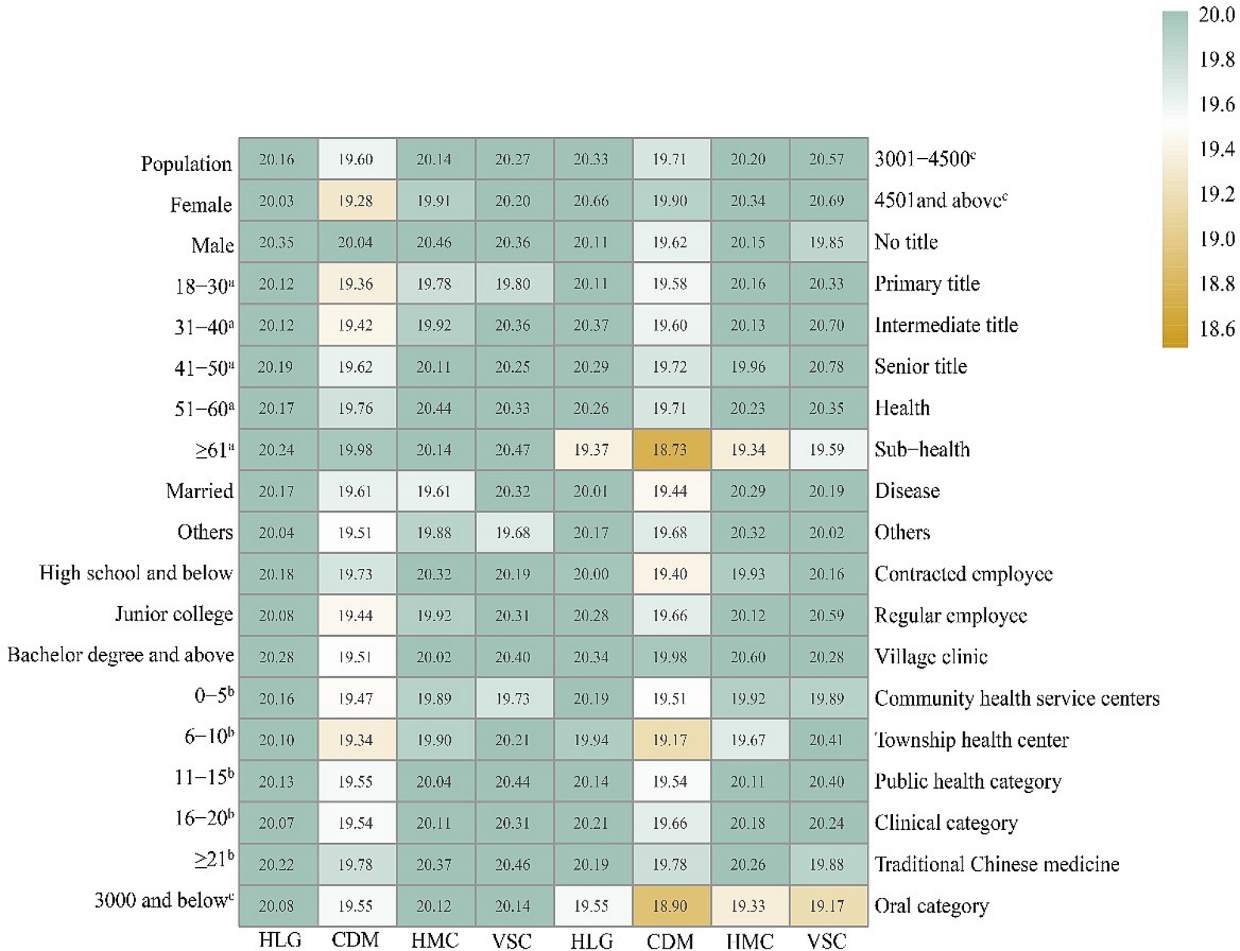
Heatmap of four dimensions service capability score. Note: HLG (healthy lifestyle guidance); CDM (chronic disease management); HMC (health management of special populations); VSC (Vaccination service capability); a for age; b for working years; c for average monthly salary

The mean scores for all four dimensions are clustered around 20, with 20.16 points for health lifestyle guidance, 19.60 points for chronic disease management, 20.14 points for health management of special population, and 20.27 points for vaccination service. Chronic disease management had the lowest score, and sub-health and oral category was significantly lower than other subgroups in four dimensions (Fig. 2). There were significant differences in the scores for the four dimensions in terms of gender, health status, employment form and type of work (all *p* < 0.05). (Supplemental file 2)

### Factors influencing public health service capability of primary healthcare personnel

The multiple linear regression analysis showed that male public health service capability scored higher than female (*P < 0.001*, 95%CI: 0.82–1.96). The participants with a bachelor’s degree scored higher than those with high school education or higher (*P = 0.019*, 95%CI: 0.18– 2.01). In comparison to average monthly salary of less than 3000 yuan, those with 3001–4500 yuan or above had higher public health service capability scores (*P* < 0.001, 95%CI: 0.79–2.05; *P* = 0.003, 95%CI: 0.72–3.44). Those with intermediate and senior professional title had higher scores for public health service capability than those without professional title (*P = 0.005*, 95%CI: 0.35–1.97; *P* = 0.010, 95%CI: 0.47–3.48). The sub-health population had lower scores of public health services than the healthy population *(P < 0.001*, 95%CI: -4.38– -2.70); The regular employees had higher public health service scores than other public health personnel (*P = 0.004*, 95%CI: -2.59–0.08). Those working in village clinics had higher scores (*P = 0.002*, 95%CI: -5.63– -1.30) than their counterparts. Those in public health, clinical, and traditional Chinese medicine scored higher than those in oral medicine (*P = 0.006*, 95%CI: 0.91–5.55; *P* = 0.007, 95%CI: 0.87–5.53; *P* = 0.034, 95%CI: 0.20–5.01). Age, marital status and working years had no effect on the public health service capability of primary healthcare personnel (*P = 0.914*, 95%CI: -1.17–1.05; *P* = 0.225, 95%CI: -2.01–0.47; *P* = 0.143, 95%CI: *P* = 0.402, 95%CI: -2.49–0.99; *P* = 0.705, 95%CI: -0.70–1.04; *P* = 0.863, 95%CI: -0.84–1.00; *P* = 0.572, 95%CI: -0.76–1.37; *P = 0,543*, 95%CI: -1.43–0.75; *P* = 0.590, 95%CI: -0.72–1.25). See Table 2 for details.

**Table 2:** Factors influencing public health service capability of primary healthcare personnel

Variables	β (95%CI)	*P*- value	aβ (95%CI) *	*P*- value		
Gender						
Female	Reference		Reference			
Male	1.80 (1.29–2.31)	< 0.001	1.39 (0.82–1.96)	< 0.001		
Age						
18–30	Reference		Reference			
31–40	0.74 (0.20–1.69)	0.123	0.06 (-1.17–1.05)	0.914		
41–50	1.11 (0.20–2.01)	0.017	0.77 (-2.01–0.47)	0.225		
51–60	1.64 (0.70–2.58)	< 0.001	1.05 (-2.44–0.35)	0.143		
≥ 61	2.30 (0.91–3.62)	< 0.001	0.75 (-2.49–0.99)	0.402		
Marital status						
Married	Reference		Reference			
Others	0.64 (-0.13–1.42)	0.105	0.17 (-0.70–1.04)	0.705		
Education level						
High school and below	Reference		Reference			
Junior college	-0.68 (-1.25– -0.11)	0.020	0.60 (-0.7–1.28)	0.080		
Bachelor degree and above	-0.21 (-0.93–0.50)	0.558	1.06 (0.18– 2.01)	0.019		
Working years						
0–5	Reference		Reference			
6–10	0.30 (-0.57–1.17)	0.502	0.08 (-0.84–1.00)	0.863		
11–15	0.90 (-0.07–1.886)	0.069	0.31 (-0.76–1.37)	0.572		
16–20	0.79 (-0.17–1.74)	0.106	0.34 (-1.43–0.75)	0.543		
≥ 21	1.58 (0.88–2.28)	< 0.001	0.27 (-0.72–1.25)	0.590		
Average monthly salary						
3000 and below	Reference		Reference			
3001–4500	0.92 (0.33–1.51)	0.002	1.42 (0.79–2.05)	< 0.001		
4501 and above	1.71 (0.39–3.02)	0.011	2.0 (0.72–3.44)	0.003		
Professional title						
No title	Reference		Reference			
Primary title	0.47 (-0.12–1.05)	0.119	0.57 (-0.23–1.17)	0.059		
Intermediate title	1.08 (0.33–1.82)	0.004	1.16 (0.35–1.97)	0.005		
Senior title	1.03 (-0.39–2.43)	0.155	1.98 (0.47–3.48)	0.010		
Health status						
Health	Reference		Reference			
Sub-health	-3.52 (-4.37– -2.68)	< 0.001	3.54 (-4.38– -2.70)	< 0.001		
Disease	-0.62 (-1.95–0.70)	0.357	1.26 (-2.59–0.07)	0.065		
Form of employment						
Others	Reference		Reference			
Contracted employee	-0.69 (-1.33–-0.04)	0.038	0.25 (-0.47–0.96)	0.501		
Regular employee	0.46 (-0.13–1.05)	0.124	1.00 (-2.59–0.08)	0.004		
Type of work unit						
Community health service centers (stations)	Reference		Reference			
Village clinic	-3.32 (-5.47– -1.18)	0.002	3.46 (-5.63– -1.30)	0.002		
Township health center	-1.52 (-2.30– -0.75)	< 0.001	2.70 (-3.75– -1.64)	< 0.001		
Category of practicing physician	-2.01 (-2.56– -1.46)	< 0.001	3.34 (-4.15– -2.52)	< 0.001		
Oral category	Reference		Reference			
Public health category	3.24 (0.92–5.56)	0.006	3.23 (0.91–5.55)	0.006		
Clinical category	3.34 (1.00– 5.67)	0.005	3.20 (0.87–5.53)	0.007		
Traditional Chinese medicine category	3.16 (0.75–5.56)	0.010	2.60 (0.20–5.01)	0.034		

95%CI refers to 95% confidence interval

*β*: Unadjusted variables. aβ*: Adjustment for gender, age, marital status, education level, working years, average monthly salary, professional title, health status, forms of employment, type of work unit, public health category

### Differences in public health service capability between urban and rural primary healthcare personnel

We selected rural primary healthcare personnel as the matched group and urban primary healthcare personnel as the control group. We employed a 1:1 nearest neighbor matching method with a caliper value of 0.03, incorporating the previously mentioned control variables as covariates. There were 1,825 (15.3%) healthcare personnel from community health service centers and 10,100 (84.7%) were from township hospitals or village clinics. Before PSM analysis, there were statistically significant differences in gender, marital status, education level, working years, average monthly salary, professional title, employment form and type of work unit between urban and rural healthcare personnel (all *P* < 0.05). The public health service capability of primary healthcare personnel in urban areas was 79.50 ± 0.33, while in rural areas was 80.30 ± 0.14. The difference between these two groups was statistically significant (*P = 0.042*), with rural personnel demonstrating higher scores than their urban counterparts.

**Fig. 3:**
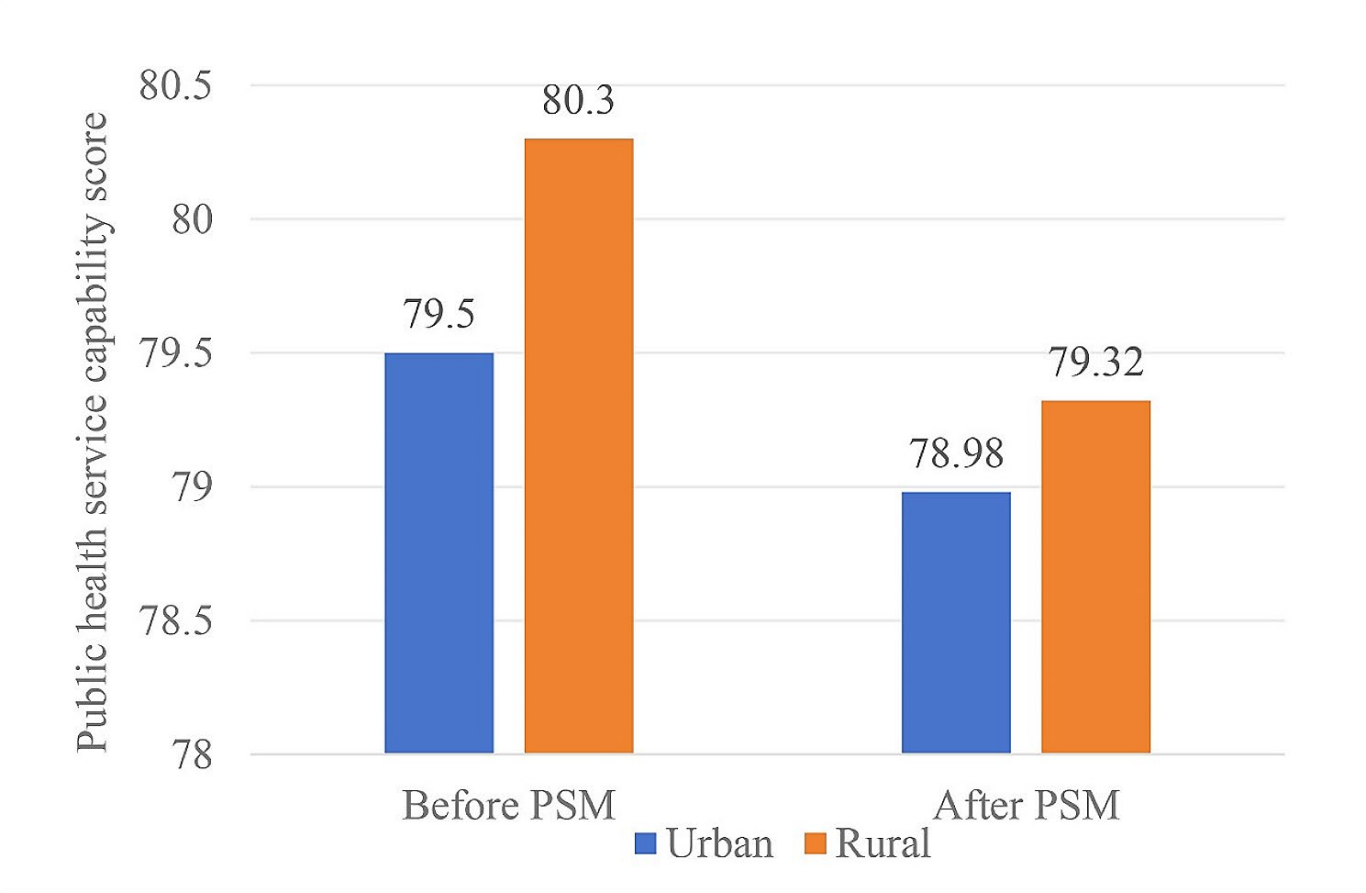
Changes of public health service capability scores in urban and rural before and after PSM

Using PSM, we matched the basic information of urban and rural healthcare personnel at a 1:1 ratio, and successfully matched 1684 pairs of subjects, with a total of 3,368 participants. After PSM, there was no statistically significant difference in the basic characteristics (all *P* > 0.05; Table 3**)**. The findings indicate that the sample achieved a good balance, effectively mitigating the influence of covariates that could have contributed to the disparity in public health service capability between urban and rural primary healthcare personnel. The public health service capability scores of urban and rural primary healthcare personnel were (78.98 ± 0.36) and (79.32 ± 0.34), respectively. After PAM, the difference remained statistically significant (*P = 0.025*), with rural areas continuing to exhibit higher public health service capacities than urban areas (Fig. 3).

**Table 3:** Comparison of the characteristics of urban and rural primary healthcare personnel before and after PSM

Variables	Before/after PSM	Urban	Rural	Standard error (%)	Error reductio (%)	t		*p*>|t|
Gender	Before	0.470	0.157	71.6			25.58	< 0.001
	after	0.167	0.169	-0.5	99.2		-0.18	0.854
Marital status	Before	1.990	1.879	30.8			13.39	< 0.001
	after	1.885	1.889	-1	96.8		-0.27	0.788
Education level	Before	2.503	3.194	-90.1			-35.32	< 0.001
	after	3.141	3.130	14.5	83.9		4.26	0.342
Working years	Before	3.660	2.385	84.5			32.77	< 0.001
	after	2.287	2.354	-11.1	86.9		-3.39	0.001
Average monthly salary	Before	1.292	1.539	-39.2			-16.89	0.007
	after	1.532	1.470	9.9	74.7		2.72	0.077
Professional title	Before	0.906	1.149	-31.1			-12	< 0.001
	after	1.140	1.108	4	87		1.17	0.242
Health status	Before	0.210	0.178	7			1.99	0.046
	after	0.175	0.178	-0.6	-1092.3		-0.22	0.822
Form of employment	Before	0.984	1.108	-15.7			-5.7	< 0.001
	after	1.156	1.111	5.6	64.2		1.71	0.087
Type of work unit	Before	2.712	2.787	-5.4			-2.13	0.033
	after	2.677	2.756	-5.7	-4.9		-1.63	0.102

## Discussion

### The welfare benefits of primary public healthcare personnel are limited

Over 50% of the elderly are working in public health services, nearly half of employees have accumulated more than twenty working years, and approximately one-third is regular employee. Most primary healthcare personnel earned less than 3,000 yuan a month. In addition, less than one-fifth of the primary healthcare personnel had bachelor’s degree or above, and few had senior professional titles. These findings indicate that the majority of primary healthcare personnel face challenges associated with a being older [29, 30], limited authorized strength, lower income and education level. Studies have shown that due to the limited number of senior professional titles, the number of promotion opportunities for healthcare personnel is limited in primary medical institutions, which seriously weakens the willingness of primary health personnel to serve [31]. Moreover, studies have shown that primary healthcare personnel will have high turnover due to heavy workload, low income and low sense of achievement [32]. This has a great impact on the quantity and quality of basic public health services in primary areas.

Hence, it becomes imperative to facilitate the rejuvenation of primary healthcare personnel by introducing fresh talent and enhancing welfare benefits [33, 34] as a measure to mitigate the attrition of skilled professionals. It is imperative to optimize the talent recruitment mechanism of primary health institutions, augment the number of professional healthcare personnel, and attract more outstanding undergraduate and postgraduate graduates to serve the primary areas and public health service. By offering attractive welfare for high educational talent, it can maintain the stability of primary healthcare personnel, and ultimately curb the attrition of talent.

### The public health service capability of primary healthcare personnel is not balanced

The average scores of the healthy lifestyle guidance capability, chronic disease management capability, health management capability of special population, and vaccination service capability were all about 20 points. This shows that most primary healthcare personnel had a good grasp of these public health service capability projects. However, the capability of chronic disease management was relatively poor among the four capabilities [35], followed by the health management capability of special population. This indicates that primary healthcare personnel still need to strengthen the training of this aspect. Research has indicated that the effective management of chronic diseases, such as pregnant women and individuals with severe mental disorders, as well as initiatives targeting special population health management, require a high degree of professional expertise and it takes years to cultivate professionals in these areas [35, 36].

Limited by the scarcity of health resources and the existence of manifold patients and various chronic diseases in primary areas, primary prevention is about improving the capability of chronic disease management and health management of special populations [37, 38], so as to protect the life and health of the population. Therefore, implementing a scientifically sound and effective training is also a crucial step in enhancing the knowledge and capability of primary healthcare service personnel. Primary health institutions should consider providing targeted training for the weak areas of primary healthcare personnel, such as chronic disease management capability and special groups health management capability. Strengthening the links between primary medical institutions and higher-level hospitals may increase the clinical knowledge and skills of healthcare personnel, which are relatively insufficient.

### The public health service capability of primary healthcare personnel is affected by many factors

The educational attainment of primary healthcare personnel in Henan Province is generally low. Less than one-fifth of these personnel hold a bachelor’s degree or higher, and even fewer possess a graduate degree or above. This underscores a significant deficiency of highly educated professionals in the extensive primary regions [39]. In addition, this study found that those with higher professional titles and working units in village clinics had better public health service capability. This could be attributed to the fact that these primary healthcare personnel often handle a substantial workload [40]. As a result, they gain more experience and attain a higher level of proficiency in public health services. This study also found that the primary healthcare personnel who were hired by regular employees and whose practice categories were clinical medicine, traditional Chinese medicine and public health had stronger public health capability. These public healthcare personnel will also enhance their knowledge and theory reserve [41]. As a reward and incentive system, the average monthly salary can directly affect the work enthusiasm of primary healthcare personnel [17] in their work.

An appropriate assessment mechanism for primary health institutions can be established to break the “ceiling effect”, overcome the limitation of performance-based salary cap. Therefore, a mechanism can be established to adjust the salary synchronously with the workload increase of primary healthcare personnel.

### There are differences in public health service capability between urban and rural primary healthcare personnel

The outcomes of the PSM analysis indicated that the service capability of rural primary healthcare personnel was slightly higher than their urban counterparts. This underscores the unequal distribution of public health service resources between urban and rural areas, potentially stemming from deficiencies in financial and human resources within rural public health services, as well as the absence of modern equipment and technology [42]. Urban primary services were more adequate in terms of capital, human resources, technology, etc., which cause populations tend to choose the superior hospital. Hence, urban primary healthcare personnel got less training in public health service. In contrast, rural primary public health institutions were the most important approaches of accessing to health services in rural areas.

Considering the influencing factors specific to region, it is essential to adopt suitable strategies and measures to foster equilibrium in public health services between urban and rural areas. In addition, attention should also be paid to the construction of professional personnel to enhance the service capability of primary healthcare personnel. Therefore, the government may also consider facilitating collaboration [43] between urban and rural public health services, through the adoption of internet technology [44] and the establishment of a consortium for urban and rural health services.

### Strength and limitation of the study

This study has the following advantages: first, the current study marks a pioneering effort by focusing on primary healthcare personnel, breaking away from the previous convention of primarily investigating public health service capabilities at the institutional levels of healthcare. Second, it is the first large-scale survey in Henan province, which can truly reflect the current public health service capability of primary healthcare personnel in Henan Province. Third, PSM was deployed to effectively control for confounding variables and to reveal the net difference in public health service capability between urban and rural areas. However, the study also has some limitations. First, it relied on self-reported survey data, which may have introduced some bias. Secondly, it used PSM with fewer variables than the total number of sets available. Thirdly, due to the cross-sectional design of this study, it could not examine causality.

## Conclusion

Primary healthcare personnel in Henan have above-average public health service capability. This approach will expedite efforts to achieve universal coverage of public health service capability and foster the equalization of public health services between urban and rural areas. A systematic, rational, and precisely targeted training approach should be implemented for individuals with lower education levels, lower mean monthly income, lower professional titles, and contracted employees. Moreover, the number of primary health professionals should be increased, and the professional title evaluation and promotion system should be improved to enhance the stability of primary public health team construction. These measures can greatly reduce the loss of professionals, improve the public health service capability of primary healthcare personnel, and guard the first line of defense for population health.

### Electronic supplementary material

Below is the link to the electronic supplementary material.

Supplementary Material 1

Supplementary Material 2

## Data Availability

The datasets used and/or analyzed during the current study are available from the corresponding author on reasonable request.
