# Supplementary material for: Assessing public health service capability of primary healthcare personnel: a large-scale survey in Henan Province, China
Source: BMC Health Serv Res. 2024 May 14;24:627. doi: 10.1186/s12913-024-11070-4 (PMC11094852; doi:10.1186/s12913-024-11070-4)
Supplement: Supplementary file 1 — Supplementary Material 1 [file 12913_2024_11070_MOESM1_ESM.docx]

**^Questionnaire Code: _____^**

**A questionnaire about public health service capability of primary healthcare personnel**

Region： Province City County /district town and villages(community)

Time： Year Month Date

Hello! Sincerely thank you for taking time out of your busy schedule to fill out this questionnaire!

The purpose of this questionnaire is to understand the public health service capability of primary healthcare personnel. Please read it carefully and truly choose the options that match your situation. Please answer the questions one by one and do not miss questions. There is no right or wrong choice for each question in the questionnaire, and it is only used for research and analysis. The questionnaire is anonymous, and your basic personal information and answers to various questions will not be named and disclosed. It is absolutely confidential. Please rest assured.

| **一、Basic characteristics (Please tick “√” or fill in according to your personal situation)** | |
| --- | --- |
| 1. Gender: ①Male ②Female 2. Age: | |
| 3. Marital status: ①Unmarried ②Married ③Divorce ④Widow/Widower | |
| 4. Politics status: | 5. Health status: ①Health ②Sub-health ③Disease (Chronic diseases and so on） |
| 6. Employment form:  ①Regular employee ②Contracted employee ③Others | |
| 7. Working years under this unit: year（More than 6 months but less than 1 year are counted as 1 year） | |
| 8. Education level: ①High school and below ②Technical secondary school/technical secondary school ③Junior College ④Undergraduate ⑤Postgraduate and above | |
| 9. Type of work unit: ①Community health service centers ②Village clinic ③Township health centers ④Community health service stations ⑤Others | |
| 10. Qualification of practice: ①Practicing physician ②Practicing assistant physician ③Township practicing assistant physician ④Township general practicing assistant physician ⑤Nurse ⑥Medical technology ⑦Others | |
| 11. Your category of practicing (assistant) physician:  ①Clinical category ②Traditional Chinese medicine category ③Oral category ④Public health category | |
| 12 Professional title:   1. Primary title ②Intermediate title ③Senior title ④No title | |
| 13. Average monthly salary (Real salary + Bonus):  ①3000 and below ②3001~4500 ③4501~6000 ④6001~7500 ⑤7501 and above | |

| **Public Health Capability**（Please tick “√” in the options that matches your personal situation） | | | | | |
| --- | --- | --- | --- | --- | --- |
| **Questions** | Strongly disagree | Disagree | Neutral | Agree | Strongly agree |
| **Healthy lifestyle guidance capability** | | | | | |
| 1. I have the capability to give dietary guidance to the residents in the area | 1 | 2 | 3 | 4 | 5 |
| 2. I have the capability to give exercise guidance to the residents in the area | 1 | 2 | 3 | 4 | 5 |
| 3. I have the capability to give decompression guidance to the residents in the area | 1 | 2 | 3 | 4 | 5 |
| 4. I have the capability to give weight loss guidance to the residents in the area | 1 | 2 | 3 | 4 | 5 |
| 5. I have the capability to give smoking cessation and alcohol limit guidance to the residents in the area | 1 | 2 | 3 | 4 | 5 |
| **Chronic disease management capability** | | | | | |
| 1. I have the capability of early screening for chronic disease | 1 | 2 | 3 | 4 | 5 |
| 2. I have the capability to predict chronic disease risk | 1 | 2 | 3 | 4 | 5 |
| 3. I have the capability of early warning and comprehensive intervention of chronic disease | 1 | 2 | 3 | 4 | 5 |
| 4. I have the capability of integrated management of chronic patient group | 1 | 2 | 3 | 4 | 5 |
| 5. I have the capability to evaluate the effect of integrated management of chronic disease | 1 | 2 | 3 | 4 | 5 |
| **Health management capability of special populations**  (Including children aged 0-6 years, pregnant women, the elderly, patients with hypertension, type 2 diabetes, severe mental disorders, tuberculosis, etc.) | | | | | |
| 1. I have received professional and technical training in special populations health management | 1 | 2 | 3 | 4 | 5 |
| 2. I mastery demographic information on special populations in the area | 1 | 2 | 3 | 4 | 5 |
| 3. I pay attention to the health education of special populations, inform the service content, and improve the willingness of health management | 1 | 2 | 3 | 4 | 5 |
| 4. I take the initiative to contact special populations, and ensure the continuity of health management | 1 | 2 | 3 | 4 | 5 |
| 5. After health management, I will timely record the relevant information in the health record to ensure the standardization of health management | 1 | 2 | 3 | 4 | 5 |
| **Vaccination service capability** | | | | | |
| 1. I am qualified to practice physician and have passed professional training in vaccination | 1 | 2 | 3 | 4 | 5 |
| 2. I understand the national immunization program policies, laws and regulations | 1 | 2 | 3 | 4 | 5 |
| 3. I understand the vaccination procedure | 1 | 2 | 3 | 4 | 5 |
| 4. I understand the types and alternatives of Category 1 and Category 2 vaccines | 1 | 2 | 3 | 4 | 5 |
| 5. I understand the safety of vaccination | 1 | 2 | 3 | 4 | 5 |
