# Supplementary material for: Assessing public health service capability of primary healthcare personnel: a large-scale survey in Henan Province, China
Source: BMC Health Serv Res. 2024 May 14;24:627. doi: 10.1186/s12913-024-11070-4 (PMC11094852; doi:10.1186/s12913-024-11070-4)
Supplement: Supplementary file 2 — Supplementary Material 2 [file 12913_2024_11070_MOESM2_ESM.docx]

Supplementary Materials

**Supplemental Table 2** The score of public health service capability of primary healthcare personnel in Henan province

| **Supplemental Table 2** The score of public health service capability of primary healthcare personnel in Henan province | | | | | | | | | | | |
| --- | --- | --- | --- | --- | --- | --- | --- | --- | --- | --- | --- |
| Variables | | Healthy lifestyle guidance capability score | | Chronic disease management capability score | | Health management capability of special populations | | Vaccination service capability score | | Public health service capability score | |
|  |  | *M* ± *SD* | P | M ± SD | P | M ± SD | P | M ± SD | P | M ± SD | P |
| Population |  | 20.16±3.81 |  | 19.60±3.99 |  | 20.14±3.88 |  | 20.27±4.04 |  | 80.17±14.12 |  |
| Gender | Female | 20.03±3.83 | <0.001† | 19.28±4.04 | <0.001† | 19.91±3.91 | <0.001† | 20.20±4.13 | 0.025† | 79.41±14.00 | <0.001† |
|  | Male | 20.35±3.79 |  | 20.04±3.87 |  | 20.46±3.79 |  | 20.36±3.92 |  | 81.21±14.21 |  |
| Age | 18-30 | 20.12±4.16 | 0.091‡ | 19.36±4.45 | <0.001‡ | 19.78±4.20 | <0.001‡ | 19.80±4.67 | <0.001‡ | 79.07±15.75 | 0.001‡ |
|  | 31-40 | 20.12±3.91 |  | 19.42±4.18 |  | 19.92±4.00 |  | 20.36±4.22 |  | 79.81±14.36 |  |
|  | 41-50 | 20.19±3.72 |  | 19.62±3.90 |  | 20.11±3.80 |  | 20.25±4.00 |  | 80.17±13.87 |  |
|  | 51-60 | 20.17±3.69 |  | 19.76±3.74 |  | 20.44±3.68 |  | 20.33±3.72 |  | 80.71±13.43 |  |
|  | ≥61 | 20.24±3.81 |  | 19.98±3.86 |  | 20.14±3.87 |  | 20.47±4.04 |  | 81.17±14.11 |  |
| Marital status | Married | 20.17±3.79 | 0.334† | 19.61±3.97 | 0.483† | 19.61±3.97 | 0.044† | 20.32±3.99 | <0.001† | 80.27±14.00 | 0.026‡ |
|  | Others | 20.04±4.08 |  | 19.51±4.29 |  | 19.88±4.16 |  | 19.68±4.54 |  | 79.11±15.42 |  |
| Education level | High school and below | 20.18±3.72 | 0.168‡ | 19.73±3.84 | 0.001‡ | 20.32±3.75 | <0.001‡ | 20.19±3.84 | 0.095‡ | 80.42±13.88 | 0.066‡ |
|  | Junior college | 20.08±3.86 |  | 19.44±4.12 |  | 19.92±3.95 |  | 20.31±4.17 |  | 79.75±14.29 |  |
|  | Bachelor degree and above | 20.28±4.00 |  | 19.51±4.21 |  | 20.02±4.05 |  | 20.40±4.04 |  | 80.21±14.50 |  |
| Working years | 0-5 | 20.16±3.95 | 0.631‡ | 19.47±4.20 | <0.001‡ | 19.89±3.95 | <0.001‡ | 19.73±4.40 | <0.001‡ | 79.25±14.92 | <0.001‡ |
|  | 6-10 | 20.10±3.92 |  | 19.34±4.17 |  | 19.90±4.00 |  | 20.21±4.30 |  | 79.55±14.47 |  |
|  | 11-15 | 20.13±3.87 |  | 19.55±4.09 |  | 20.04±4.00 |  | 20.44±4.07 |  | 80.15±14.19 |  |
|  | 16-20 | 20.07±3.79 |  | 19.54±3.99 |  | 20.11±3.82 |  | 20.31±3.88 |  | 80.04±13.88 |  |
|  | ≥21 | 20.22±3.71 |  | 19.78±3.80 |  | 20.37±3.74 |  | 20.46±3.80 |  | 80.83±13.65 |  |
| Average monthly salary | 3000 and below | 20.08±3.81 | <0.001 | 19.55±3.96 | 0.045 | 20.12±3.84 | 0.320 | 20.14±4.00 | <0.001 | 79.88±14.14 | 0.001 |
|  | 3001-4500 | 20.33±3.84 |  | 19.71±4.13 |  | 20.20±3.94 |  | 20.57±4.12 |  | 80.80±14.18 |  |
|  | 4501and above | 20.66±3.69 |  | 19.90±3.75 |  | 20.34±3.86 |  | 20.69±4.25 |  | 81.59±12.98 |  |
| Professional title | No title | 20.11±3.71 | 0.034‡ | 19.62±3.92 | 0.911‡ | 20.15±3.77 | 0.785‡ | 19.85±4.03 | <0.001‡ | 79.72±14.06 | 0.031‡ |
|  | Primary title | 20.11±3.86 |  | 19.58±4.00 |  | 20.16±3.87 |  | 20.33±4.01 |  | 80.19±14.20 |  |
|  | Intermediate title | 20.37±3.82 |  | 19.60±4.06 |  | 20.13±3.97 |  | 20.70±4.05 |  | 80.80±13.83 |  |
|  | Senior title | 20.29±4.04 |  | 19.72±4.16 |  | 19.96±4.18 |  | 20.78±4.22 |  | 80.75±14.94 |  |
| Health status | Health | 20.26±3.82 | <0.001‡ | 19.71±3.99 | <0.001‡ | 20.23±3.85 | <0.001‡ | 20.35±4.03 | <0.001‡ | 80.55±14.14 | <0.001‡ |
|  | Sub-health | 19.37±3.77 |  | 18.73±3.99 |  | 19.34±4.00 |  | 19.59±4.17 |  | 77.03±13.84 |  |
|  | Disease (Chronic disease, etc.) | 20.01±3.58 |  | 19.44±3.82 |  | 20.29±3.69 |  | 20.193±.78 |  | 79.93±13.30 |  |
| Form of employment | Others | 20.17±3.66 | 0.008‡ | 19.68±3.77 | 0.005‡ | 20.32±3.65 | <0.001‡ | 20.02±3.84 | <0.001‡ | 80.18±13.62 | 0.002‡ |
|  | Contracted employee | 20.00±3.92 |  | 19.40±4.13 |  | 19.93±3.94 |  | 20.16±4.21 |  | 79.50±14.41 |  |
|  | Regular employee | 20.28±3.88 |  | 19.66±4.10 |  | 20.12±4.01 |  | 20.59±4.09 |  | 80.65±14.37 |  |
| Type of work unit | Village clinic | 20.34±3.61 | <0.001‡ | 19.98±3.66 | <0.001‡ | 20.60±3.56 | <0.001‡ | 20.28±3.64 | <0.001‡ | 81.19±13.33 | <0.001‡ |
|  | Community health service centers (stations) | 20.19±3.84 |  | 19.51±4.02 |  | 19.92±3.92 |  | 19.89±4.45 |  | 79.50±14.07 |  |
|  | Township health center | 19.94±4.03 |  | 19.17±4.31 |  | 19.67±4.14 |  | 20.41±4.33 |  | 79.18±14.98 |  |
| Category of practicing (assistant) physician | Public health category | 20.14±3.85 | 0.194‡ | 19.54±4.06 | 0.020‡ | 20.11±3.90 | 0.034‡ | 20.40±4.00 | <0.001‡ | 80.19±14.26 | 0.049‡ |
|  | Clinical category | 20.21±3.76 |  | 19.66±3.94 |  | 20.18±3.84 |  | 20.24±4.06 |  | 80.29±13.85 |  |
|  | Traditional Chinese medicine category | 20.19±3.76 |  | 19.78±3.82 |  | 20.26±3.75 |  | 19.88±4.06 |  | 80.11±14.08 |  |
|  | Oral category | 19.55±4.17 |  | 18.90±4.18 |  | 19.33±4.15 |  | 19.17±4.60 |  | 76.95±15.38 |  |

P† value from t-test; P‡ value from analysis of variance.
